# Severe Fever with Thrombocytopenia Syndrome, Shandong Province, China, 2011

**DOI:** 10.3201/eid2001.120532

**Published:** 2014-01

**Authors:** Hong-Ling Wen, Li Zhao, Shenyong Zhai, Yuanyuan Chi, Feng Cui, Dongxu Wang, Ling Wang, Zhiyu Wang, Qian Wang, Shoufeng Zhang, Yan Liu, Hao Yu, Xue-Jie Yu

**Affiliations:** Shandong University School of Public Health, Jinan, China (H.-L. Wen. L. Zhao, Y. Chi, D. Wang, Z. Wang, X.-J. Yu);; Zibo Municipal Center for Disease Control and Prevention, Zibo, China (S. Zhai, F. Cui, L. Wang);; Yiyuan County Center for Disease Control and Prevention, Yiyuan, China (Q. Wang, S. Zhang);; University of Texas Medical Branch, Galveston, Texas, USA (Y. Liu, H. Yu, X.-J. Yu)

**Keywords:** severe fever with thrombocytopenia syndrome, SFTS, severe fever with thrombocytopenia syndrome virus, SFTSV, viruses, bunyavirus, epidemiology, clinical characteristics, laboratory characteristics, Shandong Province, China

## Abstract

Severe fever with thrombocytopenia syndrome (SFTS) is an emerging infectious disease in China. The incidence and clinical and laboratory characteristics of SFTS are not clearly defined. During May 22–October 2, 2011, a total of 24 patients with fever, thrombocytopenia, and leukopenia were clinically diagnosed as having SFTS in Yiyuan County, Shandong Province, China. We conducted laboratory tests for these SFTS patients. SFTS virus (SFTSV) infection was confirmed in 22 patients by using reverse transcription PCR and ELISA by acute-phase and convalescent-phase serum samples. Clinical and laboratory manifestations included fever (100%), gastrointestinal symptoms (91%), myalgia (55%), chills (41%), thrombocytopenia (100%), and leukopenia (95%).

## Abstract 2

Supportive therapy is recommended before laboratory confirmation of this disease.

Severe fever with thrombocytopenia syndrome (SFTS) is an emerging infectious disease that was identified in 2009 in rural areas in China. This disease is caused by SFTS virus (SFTSV), a novel bunyavirus in the family *Bunyaviridae*, genus *Phlebovirus*. (*1*). Fatal cases of infection with SFTSV have been recently reported in Japan and South Korea (*2**,**3*).

SFTS is a severe disease and has had a case-fatality rate of 12%–30% in China (*1*). The major manifestations of SFTS are fever, thrombocytopenia, leukopenia, and increased serum levels of hepatic aminotransferases. SFTSV has been detected in ticks and might be transmitted by them (*1**,**4*). Occasionally, the disease can also be transmitted from person to person through contact with infected blood or mucus (*5**–**9*).

The epidemiologic and clinical characteristics of SFTSV infection are not well defined. Approximately 30% of clinically diagnosed cases of SFTS cannot be confirmed by laboratory tests (*1**,**10*), and clinicians may confuse this disease with diseases caused by other pathogens. Therefore, to obtain information on clinical and laboratory characteristics of this disease, with a focus on diagnosis, we used acute-phase and convalescent-phase serum samples from 24 patients given a clinical diagnosis of SFTS in Yiyuan County, Shandong Province, China, an area to which SFTSV is endemic (*11*), 

## Study Site

**Figure 1:**
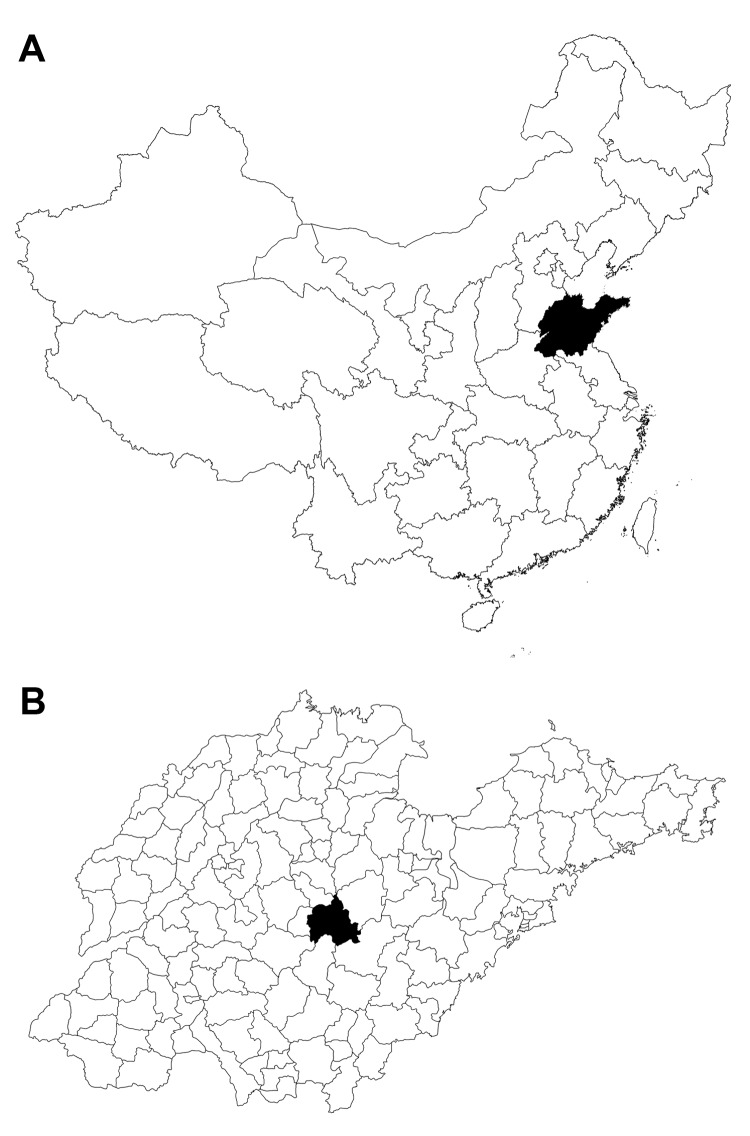
A) Shandong Province, China (black area) where severe fever with thrombocytopenia syndrome was studied, 2011. B) Yiyuan County (black area) in Shandong Province.

Yiyuan County is located in Shandong Province in eastern China (35°55 –36°23′Ν, 117°48'–118 °31′Ε (Figure 1). It has a population of ≈550,000 persons, of whom 85% live in rural areas.

## Clinical Case Definition and Blood Collection

We defined a clinically diagnosed case-patient with SFTS as a patient who had fever, leukopenia, or thrombocytopenia without another known acute infectious disease. We did not have data on what other infectious diseases were ruled out. We defined a laboratory-confirmed case of SFTS as a clinically diagnosed case with a positive antibody or reverse transcription PCR (RT-PCR) result for SFTSV. Acute-phase serum samples, clinical information, and laboratory data for all patients given a diagnosis of SFTS in 2011 in Yiyuan County were submitted to the Yiyuan County Centers for Disease Control and Prevention.

Acute-phase serum samples were obtained 4–13 days after onset of illness. Sixteen samples were obtained during the first week, and the remaining samples were obtained during the second week. Convalescent-phase serum samples were obtained 3–6 months after patients had recovered from the disease. All patients were admitted to Yiyuan County People’s Hospital, the only hospital in Yiyuan County. Thus, we believe that we enrolled all SFTS patients from Yiyuan County during 2011. The research protocol was approved by the human bioethics committee of Shandong University, and all participants provided written informed consent.

## Detection of Virus RNA in Acute-phase Serum Samples

Total RNA was extracted from blood by using the QIAamp Viral RNA Mini Kit (QIAGEN, Hilden, Germany). RNA was used as template for RT-PCR to amplify SFTSV RNA by using primers derived from large (L) and small (S) RNA segments of the virus (Table 1). RT-PCR was performed by using the One-Step PCR Kit (QIAGEN), and the RT-PCR product was used as template for nested PCR. Nested PCR products were sequenced to confirm SFTSV sequences.

**Table 1:** Primers for RT-PCR and nested PCR testing for severe fever with thrombocytopenia syndrome virus, Shandong Province, China, 2011*

Type of primer	Primer name	Sequence, 5′→3′	Virus segment
Primary	F1_S_	CAGCCACTTTACCCGAACAT	Small
	R1_S_	GGAAAGACGCAAAGGAGTGA	
Nested	F2_S_	CTGGTCTCTGCCCTCTCAAC	
	R2_S_	GGATTGCAGTGGAGTTTGGTG	
Primary	F1_L_	GGCAGCAAACCAGAAGAAAG	Large
	R1_L_	CATTTCTCCGAGGGCATTTA	
Nested	F2_L_	GGGTCTCCTGCTTAGCACAGG	
	R2_L_	TCAGAFAAFACCCTGCCAGT	

*RT-PCR, reverse transcription PCR; F, forward; R, reverse.

## ELISA

Serum samples were tested for antibodies (IgG and IgM) against SFTSV by using a double-antigen sandwich ELISA kit (Shanghai Zhengshuo Biotech Company, Shanghai, China) (*12**,**13*). The ELISA kit used recombinant nucleoprotein (NP) of SFTSV as antigen for coating plates. In initial screening, an undiluted serum sample was used to determine whether the sample was positive for antibodies against SFTSV. Positive serum samples were further diluted in 2-fold increments starting at 1:2. Each sample (50 μL) was added to wells of antigen-coated plates, and plates were incubated for 30 min at 37°C to enable SFTSV antibodies to bind to NP of SFTSV. The plates were washed, horseradish peroxidase–labeled recombinant SFTSV NP was added, and reactivity was detected by using substrates for horseradish peroxidase. Absorbance was read at 450 nm. A serum samples was considered to contain antibody to SFTSV when absorbance of the sample was ≥2.1 fold greater than that of a negative control (provided by the manufacturer), which was 3 SD above the mean optical density 450 nm for the person sampled. The ELISA had similar specificity and sensitivity as a microneutralization assay and showed no cross-reactivity with antibodies against hantavirus or dengue virus (*12**,**13*).

## Patients

Twenty-four patients were given a diagnosis of SFTS according to our case definition. We considered that 22 of these 24 patients were confirmed SFTS cases because blood samples from these patients were PCR positive for SFTSV RNA and/or had antibodies positive to SFTSV. Major clinical manifestations of laboratory-confirmed case-patients are shown in Table 2. The frequency of major clinical signs and symptoms in these patients was 100% (22/22) for fever, 91% (20/22) for gastrointestinal symptoms (nausea, vomiting, diarrhea, abdominal pain), 55% (12/22) for myalgia, and 46% (10/22) for lymphadenopathy. Convalescent-phase blood samples were obtained from 21 patients; 95% (20/21) had leukopenia (leukocyte count <4 × 10^9^cells/L) and 100% (21/21) had thrombocytopenia (platelet count <150 × 10^9^/L). All patients recovered from their illness.

**Table 2:** Clinical parameters for 22 patients with confirmed severe fever with thrombocytopenia syndrome, Shandong Province, China, 2011

Clinical parameter	No. (%) patients
Sign or symptom	
Fever	22 (100)
Gastrointestinal	20 (91)
Myalgia	12 (55)
Lymphadenopathy	10 (45)
Chills	9 (41)
Headache	7 (32)
Flank pain	7 (32)
Laboratory test*	
Thrombocytopenia	21 (100)
Leukopenia	20 (95)

*Blood samples were obtained from 21 patients.

## RT-PCR

SFTSV RNA L and S segments were amplified by 1-step RT-PCR and then by a nested PCR that produced a 900-bp fragment and a 600-bp fragment, respectively. The PCR detection rate was 50% (12/24) for the S segment (Table 3) and 17% for the L segment. All L segment–positive patients were also positive for S segment. PCR products were confirmed to be SFTSV RNA by DNA sequencing.

**Table 3:** Detection of SFTSV RNA and virus-specific antibody in serum samples of 24 patients, Shandong Province, China, 2011*

Patient no. or parameter	Day of blood collection after disease onset	Acute-phase serum samples, n = 24			Convalescent-phase serum samples, n = 21	RT-PCR– or ELISA–confirmed SFTSV cases, n = 24
RT-PCR, S RNA segment	ELISA titer	ELISA titer
1	5	–	2		0	+
2	10	+	64		ND	+
3	6	–	0		64	+
4	4	–	0		0	–
5	9	–	256		≥512	+
6	7	+	0		≥512	+
7	4	+†	0		128	+
8	13	–	0		128	+
9	5	–	0		0	–
10	7	+†	0		256	+
11	6	–	0		256	+
12	6	+	64		≥512	+
13	7	+†	0		≥512	+
14	5	–	256		≥512	+
15	4	+†	0		≥512	+
16	10	+	0		ND	+
17	6	+	32		128	+
18	13	–	512		NA	+
19	10	–	64		256	+
20	10	–	256		256	+
21	7	+	512		256	+
22	6	+	32		≥512	+
23	8	+	32		32	+
24	6	–	64		256	+
SFTSV positivity rate, %	NA	50	54		86	92
Sensitivity, %	NA	55	59		95	NA

* SFTSV positivity rate was calculated by using patients given a clinical diagnosis (n = 24) as the denominator. Sensitivity was calculated by using laboratory-confirmed cases (n = 22) as the denominator. SFTSV, severe fever with thrombocytopenia syndrome virus; RT-PCR, reverse transcription PCR; S, small; –, negative; +, positive; ND, not determined; NA, not applicable. †Positive by RT-PCR for S and large segments.

## Detection of Antibodies against SFTSV

ELISA showed that 54% (13/24) of acute-phase serum samples and 86% (18/21) of convalescent-phase serum samples contained antibodies against SFTSV (Table 3). Although a 4-fold increase in antibody titer was not a criterion for ELISA, we diluted serum samples to determine whether serum antibody titer changed in the patients. Results indicated that 8/21 samples showed increased antibody titers and 1/21 showed a decreased titer.

## Sensitivity of PCR and ELISA for Diagnosis of SFTSV Infection

The sensitivities of RT-PCR and ELISA were calculated by using laboratory-confirmed cases as the denominator. Sensitivities were 55% (12/22) for RT-PCR and 59% (13/22) for ELISA. By combining RT-PCR and ELISA results for acute-phase serum samples, we found that 86% (19/22) of patients were given a diagnosis of SFTSV (Table 4). Of the 22 patients infected with SFTSV, 14 had acute-phase serum samples obtained during the first week after onset of illness; 9 (64%) of 14 had SFTSV RNA detected by RT-PCR and 7 (50%) of 14 had antibodies against SFTSV detected by ELISA. Of the 8 patients who had acute-phase serum samples obtained during the second week after onset of illness, 3 (38%) of 8 had SFTSV detected by RT-PCR and 6 (75%) of 8 had antibodies against SFTSV detected by ELISA. The difference in assay performance by week of illness onset was not significant (p>0.05, by χ^2^ test).

**Table 4:** Test results for severe fever with thrombocytopenia syndrome virus–infected patients, Shandong Province, China, 2011*

Acquisition time after illness onset, wk	RT-PCR		ELISA		No. patients
	+	–	+	–	
<1	9	5	7	7	14
1−2	3	5	6	2	8

*RT- PCR, reverse transcription PCR; +, positive; –, negative.

## Epidemiology

**Figure 2:**
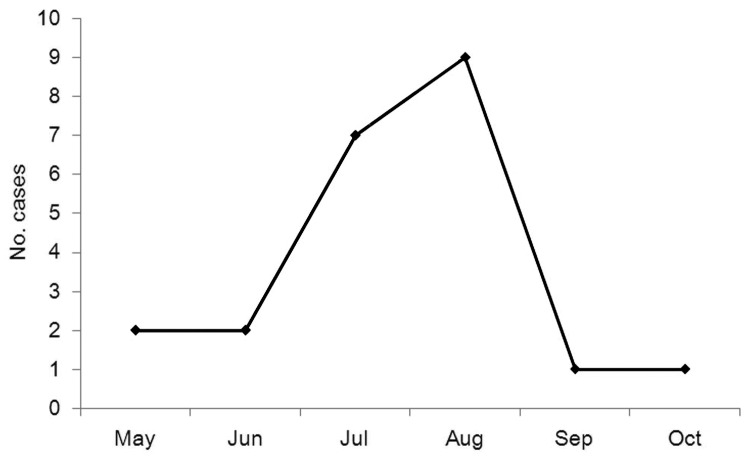
Cases of severe fever with thrombocytopenia syndrome, by month of illness onset, Shandong Province, China, 2011.

The 22 confirmed case-patients (9 women and 13 men) ranged in age from 40 to 78 years (median age 63.5 years). Patients were hospitalized during days 1–7 after onset of illness (median 4 days). The first case of SFTS occurred on May 22, and the last case occurred on October 2; 71% of the cases occurring during July–August (Figure 2). We confirmed 22 cases of SFTSV infection in Yiyuan County in 2011.

## Conclusions

Our results showed that the RT-PCR detection rate for SFTSV is higher for blood samples obtained in the first week (64%) than for those obtained in the second week (38%) after illness onset; the SFTSV antibody detection rate showed a reverse pattern. We did not observe a difference in RT-PCR detection rate of SFTSV or antibody detection rate for serum samples collected during the first and second weeks of illness. Nonetheless, our findings support use of RT-PCR with serum samples for confirming a diagnosis of SFTS during the first week after illness onset and serum ELISA for diagnosis during the second week.

There are 2 possible explanations for 2 patients having negative results for both laboratory tests. The first explanation is that levels of SFTSV virus and antibody were below detection thresholds. The second explanation is that patients were truly negative for SFTS and were ill because of infections with other pathogens.

Our study had 2 limitations. First, our sample size of reported clinical cases was small, which may have limited our ability to detect a difference between PCR and antibody detection rates during the first and second weeks of illness. Second, our clinical case definition might not have been sensitive or specific because we had access only to data that were reported by local clinicians (suspect cases reported to the Yiyuan County Centers for Disease Control and Prevention), and we did not know what specific infections were ruled out.

We obtained acute-phase serum samples from 24 patients given a clinical diagnosis of SFTS and analyzed these samples SFTSV RNA by using RT-PCR and for SFTSV antibodies by using ELISA. Cases of SFTS were confirmed for 19 (86%) of these patients when acute-phase serum samples were analyzed. Previous reports for SFTS indicated that when acute-phase serum samples were tested, ≈70% of patients with SFTS had detectable virus or virus antibodies by similar methods (*1**,**10*). Among convalescent-phase serum samples from 21 patients, 8 showed seroconversion for SFTSV. Among these 8 patients, 5 had detectable SFTSV in acute-phase serum samples. However, convalescent-phase serum samples were needed for 3/22 patients to confirm the diagnosis. It was unlikely that patients who showed seroconversion were infected after hospital discharge because of low seroprevalence of SFTSV in the study region (*11*).

Our results suggest that RT-PCR or ELISA alone is insufficiently sensitive for diagnosis of SFTSV infection in the early stage of SFTS. However, combining RT-PCR and ELISA results can increase sensitivity to 86%. RT-PCR with primers for virus S segment was more sensitive than that with primers for virus L segment, which was likely caused by primer length. We conclude that in areas to which SFTSV is endemic, patients with clinically compatible SFTS (fever, thrombocytopenia, or leukopenia without another known infectious disease) should be treated with early supportive therapy, even before laboratory confirmation is available.
